# Viral RNA Degradation Makes Urine a Challenging Specimen for Detection of Japanese Encephalitis Virus in Patients With Suspected CNS Infection

**DOI:** 10.1093/ofid/ofz048

**Published:** 2019-02-06

**Authors:** Tehmina Bharucha, Onanong Sengvilaipaseuth, Malee Seephonelee, Malavanh Vongsouvath, Manivanh Vongsouvath, Sayaphet Rattanavong, Géraldine Piorkowski, Marc Lecuit, Christopher Gorman, Jean-David Pommier, Jeremy A Garson, Paul N Newton, Xavier de Lamballerie, Audrey Dubot-Pérès

**Affiliations:** 1 Lao-Oxford-Mahosot Hospital-Wellcome Trust Research Unit (LOMWRU), Microbiology Laboratory, Mahosot Hospital, Vientiane, Lao P.D.R; 2 Division of Infection and Immunity, University College London, London, UK; 3 Nuffield Department of Clinical Medicine, Centre for Tropical Medicine and Global Health, University of Oxford, Churchill Hospital, Oxford, UK; 4 Unité des Virus Émergents (UVE: Aix-Marseille Univ – IRD 190 – Inserm 1207 – IHU Méditerranée Infection), Marseille, France; 5 Biology of Infection Unit, Inserm U1117, Institut Pasteur, Paris, France; 6 Division of Infectious Diseases and Tropical Medicine, Necker-Enfants Malades University Hospital, Paris Descartes University, Paris, France; 7 Institut Pasteur du Cambodge, Institut Pasteur International Network, Phnom Penh, Cambodia; 8 Transfusion Microbiology, NHS Blood and Transplant, London, UK

**Keywords:** diagnosis, flavivirus, Japanese encephalitis virus, RNA, RT-qPCR

## Abstract

**Background:**

Japanese encephalitis virus (JEV) is a leading cause of central nervous system (CNS) infections in Asia and results in significant morbidity and mortality. JEV RNA is rarely detected in serum or cerebrospinal fluid (CSF), and diagnosis of JEV infection is usually based on serological tests that are frequently difficult to interpret. Unlike serum or CSF, urine is relatively easy to obtain, but, to date, there has been minimal work on the feasibility of testing urine for JEV RNA.

**Methods:**

We investigated the use of lysis buffer and a Microsep device to optimize urine storage for detection of JEV RNA by reverse transcription real-time polymerase chain reaction (RT-qPCR). The best of the studied methods was then evaluated in consecutive patients admitted to the hospital with suspected CNS infections in Laos.

**Results:**

We demonstrated degradation of JEV RNA in urine after even short storage periods at 4°C or –80°C. Although there was no advantage in using a Microsep concentration device alone, immediate addition of lysis buffer to fresh urine improved the detection of JEV RNA at the limit of detection.

**Conclusions:**

In 2 studies of 41 patients with acute encephalitis syndrome, 11 (27%) were positive for JEV IgM in CSF and/or serum, and 2 (4.9%) were JEV RT-qPCR positive from throat swabs. JEV RNA was not detected in any of these patients’ urine samples. However, lysis buffer was only used during a prospective study, that is, for only 17/41 (41%) patient urine samples. Our findings suggest a need for larger studies testing urine for JEV RNA, with urine collected at different times from symptom onset, and using lysis buffer, which stabilizes RNA, for storage.

Japanese encephalitis virus (JEV) infection is a mosquito-borne disease that predominantly affects children in rural parts of Asia with poor access to health care services [1]. It is characterized by neurological complications, leading to considerable disability, mortality, and socioeconomic costs. There is no evidence for any effective treatment for JEV infection, but there are several licensed vaccines to prevent disease. Nonetheless, vaccination programs may only be appropriately implemented and evaluated if there is an accurate diagnostic test demonstrating presence of the infection [2–4].

The mainstay of diagnosis is detection of anti-JEV IgM in cerebrospinal fluid (CSF) on admission to the hospital, as the virus is rarely detected in CSF or serum by the time patients present with neurological complications [5]. Although the gold standard is a neutralization assay, this requires paired acute and convalescent samples, considerable laboratory facilities, and expertise. For this reason, the standard diagnostic test, and that recommended by the World Health Organization (WHO), is anti-JEV IgM capture enzyme-linked immunosorbent assay (MAC-ELISA) [6, 7]. However, there are increasingly recognized limitations in the use of MAC-ELISA for the diagnosis of JEV infection, with poor accuracy or translation to the field. In particular, there is low specificity related to persistence of IgM postinfection or -vaccination, cross-reactivity with other flaviviruses, and nonspecific reactivity [8].

Detection of JEV RNA is specific and informs knowledge of the molecular epidemiology of the virus. Body fluids other than serum and CSF may be suitable for detection of JEV RNA. It has recently been demonstrated that JEV RNA may be detected in throat swabs of patients with JEV confirmed by JEV MAC-ELISA in CSF [9]. Other mosquito-borne flaviviruses, such as West Nile virus, Zika virus, and dengue virus, have been detected in urine [10–13]. There have been reports of the persistence of Zika virus and West Nile virus in patients’ urine after viremia is undetectable, and the possibility of harnessing this sample type to improve the sensitivity of molecular diagnostic techniques has been proposed [10–12]. There are clear advantages to the use of urine, as it is an abundant sample that, unlike serum and CSF, does not require an invasive procedure to obtain [14, 15].

There has been minimal investigation of the use of urine to detect JEV by reverse transcription polymerase chain reaction (RT-PCR) [16]. A retrospective study of 52 patients with JEV infection suggested that JEV RNA was not detected in urine, although there have been 2 recent case reports of detection [17–19]. The aims of this study were (1) to evaluate urine storage with and without a lysis buffer, as well as concentration with a Microsep device, for subsequent detection of JEV RNA by real-time RT-PCR (RT-qPCR) and (2) to evaluate these methods in a study of consecutive patients admitted to the hospital with suspected central nervous system (CNS) infections.

## METHODS

### Evaluation of Urine Preparation and Storage

#### Preparation of JEV Dilutions in Urine

In Biosafety Level 3, JEV Laos strain JEV_CNS769_Laos_2009 (GenBank KC196115, genotype 1), isolated from the CSF of a patient with a suspected CNS infection, was cultured in Vero cells [20]. The culture supernatant (JEV isolate) was then used to prepare serial 10-fold dilutions in urine at room temperature from a healthy human volunteer for evaluation of storage conditions. Aliquots at each dilution were extracted in triplicate and tested by JEV RT-qPCR (as described below) to identify the limit of detection (LOD; last dilution positive for all 3 replicates). JEV isolate dilutions in urine were prepared at 10^2^ LOD (“high”), 10^1^ LOD (“medium”), and 10^0^ LOD (“low”).

#### Evaluation of Preparation and Storage Conditions

##### One hundred forty microliters

and 5 mL of each JEV urine dilution, “high,” “medium,” and “low,” were stored neat or mixed with an equal volume of RNA-stabilizing lysis buffer, that is, buffer AVL with 1% carrier RNA (1 μg/μL; Qiagen), each in triplicate. The aliquots were stored at 4°C for 6 hours, and then –80°C for 4 days, 14 days, and 30 days.

After return to room temperature, aliquots at each dilution, with and without buffer AVL carrier RNA, were either extracted neat or concentrated from a 5-mL dilution using a microcentrifuge device, Microsep Advance centrifugal device 100K MWCO 5 mL (VWR, Thailand). The device allows rapid concentration and purification of up to 5.0 mL of biological samples using an Omega membrane with an ultrafiltration molecular weight cutoff (MWCO) of 100K. Five milliliters was concentrated for 7 minutes at 3220*g* to a final volume of 140 µL, and if a total of 10 mL (5 mL dilution and 5 mL buffer AVL carrier RNA), then the remaining 5 mL was pipetted into the same Microsep device and concentrated for 7 minutes at 3220*g* to a final volume of 280 µL.

#### Nucleic Acid Extraction

##### One hundred forty–microliter

JEV urine aliquots were extracted using a QIAamp Viral RNA Mini Kit (Qiagen) following the manufacturer’s instruction and eluted in 60 μL. A modification was made to the protocol such that, if 140 μL of buffer AVL carrier RNA had been added before freeze-storing as a nucleic acid stabilizer (described above), then 420 μL of buffer AVL carrier RNA was added during the extraction instead of 560 μL of buffer AVL carrier RNA.

##### RT-qPCR

An in-house JEV RT-qPCR assay targeting the NS2A region, previously developed and validated [5], was used. JEV NS2A RT-qPCR was performed in duplicate using the SuperScript III One-Step RT-PCR System with Platinum Taq DNA Polymerase (Superscript-III kit; Thermo Fisher): 25-μL reaction volume; 600-nM forward (5’-AGCTGGGCCTTCTGGT-3’) and reverse (5’-CCCAAGCATCAGCACAAG-3’) primers; 300-nM probe (5’ FAM-CTTCGCAAGAGGTGGACGGCCA-TAMRA 3’) and 7.5 μL of RNA. Thermocycling conditions: 50°C for 15 minutes, 95°C for 2 minutes, 45 × [95°C for 15 seconds + 62°C for 45 seconds]. Positive (JEV RNA Genotype 3, UVE/JEV/UNK/TW/RP9-190 [GenBank KF907505, EVA 001V-02344]) and negative (no template) controls were performed for each RT-qPCR run. An internal control (10-μL MS2 phage) was added to each patient sample before extraction. Subsequently, a separate MS2 RT-qPCR run was performed to control the extraction process and to exclude inhibition, as previously described [21]. All RT-qPCR assays for optimization and subsequent experiments were run using a CFX96 qPCR detection system (Biorad Laboratories) with manual baseline and threshold settings. Cq >40 were reported as negative, and ≤40 as positive. The LOD was defined as the last dilution in which all the replicates were positive.

### Patient Samples

#### Ethics

Ethical clearance was granted by the Ethical Review Committee of the Faculty of Medical Sciences, National University of Lao and the Oxford University Tropical Ethics Research Committee, Oxford, UK. The study was performed in accordance with relevant guidelines and regulations.

#### Study Groups

##### Retrospective Study.

We performed a retrospective study of patients recruited in the South-East Asia Encephalitis (SEAe) study [22] conducted at Mahosot Hospital, Vientiane, Laos, between 2014 and 2017. CSF, serum, urine, and throat swab (using Sigma Virocult, Medicale Wire) samples at patient inclusion, along with follow-up serum at hospital discharge, were collected and stored at –80°C. Patients included in our study met all the following criteria: (1) were admitted to hospital with signs/symptoms fulfilling the SEAe clinical case definition for acute encephalitis syndrome (AES), (2) had no contraindication for lumbar puncture, (3) presented within 7 days of onset of symptoms, (4) gave written informed consent, and (5) had urine samples available for testing, collected within 24 hours of admission. The SEAe criteria included altered mental status for over 24 hours and 1 of (1) fever, (2) seizure, or (3) focal neurology.

##### Prospective Study.

A prospective study was performed of SEAe study participants admitted to Mahosot Hospital from June to December 2017, as described above except that there was no time limit for onset of symptoms. In this study, the urine storage conditions were modified, per the results of optimization experiments (see below).

#### Anti-JEV IgM ELISA (JEV MAC-ELISA)

As recommended by the WHO, all CSF and serum samples were tested using the JE Detect Kit (InBios) [23] following the manufacturer’s instructions. A 1/10 dilution was used for CSF samples, as recommended by the WHO [24].

#### RNA Extraction

##### Three hundred microliters

of urine was mixed with 100 µL of buffer ATL (Qiagen), and the total 400-µL volume extracted and eluted in 60 µL with the EZ1 Virus Mini Kit v2.0 (Qiagen, Hilden, Germany), following manufacturer’s instruction. Two hundred microliters of serum, CSF, and throat swabs were extracted and eluted in 60 µL with the same kit.

##### Real-time RT-PCR (RT-qPCR)

The same JEV NS2A RT-qPCR assay was performed, as described above; however, a 50-µL reaction volume was used to improve sensitivity, with a 15-µL sample volume, as reported [5]. An additional in-house RT-qPCR assay, targeting the NS3 region, was also performed [5]. Per extensive optimization and validation experiments previously published [5], the NS2A and NS3 assays are highly sensitive, with LODs ~4 copies per reaction. The JEV NS3 RT-qPCR was performed with SuperScript III One-Step RT-PCR System with the Platinum Taq DNA Polymerase Kit (Thermo Fisher): 50-μL reaction volume; 600-nM forward (5’-GCAATGTGYCTCCAAAGAGC-3’) and reverse (5’-GTCGATGACCCTGCTCGC-3’) primers; 300-nM probe (5’FAM-TCCTATGAYACAGAATAYCCAAA-MGB NFQ 3’); 15 μL of RNA. Thermocycling conditions: 50°C for 15 minutes; 95°C for 2 minutes; 45 × [95°C for 15 seconds + 56°C for 45 seconds]. An internal control (MS2 phage; details as above) was added to each patient sample. After extraction, MS2 RT-qPCR was performed to control the extraction process and to exclude inhibition, as previously described [21].

## RESULTS

### Evaluation of Urine Preparation and Storage

JEV isolate, extracted in duplicate and tested by JEV NS2A RT-qPCR in duplicate, had a mean Cq value of 12.18. JEV isolate was serially diluted 10-fold in urine (10^–4^ to 10^–10^); then each dilution was extracted in triplicate and submitted to JEV RT-qPCR. The LOD for JEV detection by RT-qPCR in urine was 10^–7^ (Table 1). Further experiments were performed utilizing 10^–5^ dilution as “high” with a mean Cq of 27.45, 10^–6^ dilution as “medium” with a mean Cq of 31.27, and 10^–7^ dilution as “low” with a mean Cq of 34.87. The dilutions were compared (1) with or without concentration with the Microsep device, (2) stored neat or with lysis buffer (buffer AVL carrier RNA), and (3) stored at 4°C for 6 hours, or at –80°C for 4 days, 14 days, and 30 days.

**Table 1.** JEV RT-qPCR Cq Values of 10-Fold Serial Dilutions of JEV Cell Culture Supernatant in Urine

Dilution of JEV in Urine	Extract 1	Extract 2	Extract 3	Mean Cq
10^–4^	23.71	23.40	23.23	23.44
10^–5^	27.49	27.56	27.29	27.45
10^–6^	31.10	31.47	31.22	31.27
10^–7^	35.51	35.27	33.85	34.87
10^–8^	38.12	Neg	36.67	Neg
10^–9^	Neg	Neg	35.84	Neg
10^–10^	Neg	37.47	Neg	Neg
Negative control (urine)	Neg	Neg	Neg	Neg

Extraction was performed immediately after spiking the urine and preparation of dilutions. Positive (JEV-G3 RNA) and negative (no template) controls were tested in duplicate in the same run; MS2 internal control was added to all samples before extraction, and MS2 PCR from all extracts was positive with expected Cq values. “Neg” = No Cq or Cq > 40. The limit of detection (LOD), defined here as the last dilution positive for all 3 replicates, is shaded in gray.

Abbreviations: JEV, Japanese encephalitis virus; RT-qPCR, reverse transcription real-time polymerase chain reaction.

Table 2 provides JEV RT-qPCR results for the different urine preparation and storage conditions. JEV was detected in the medium and high neat aliquots, but not in the low aliquot after storage at 4°C for 6 hours, or at –80°C for 4 days, 14 days, and 30 days. Storage in buffer AVL carrier RNA enabled detection of JEV RNA in the “low” dilution for all storage periods. Microsep centrifugation of 5 mL gave similar results without AVL; however, with AVL, the Cq was marginally lower at all dilutions tested.

**Table 2.** Results of Evaluation of Urine Preparation and Storage Conditions for the Detection of JEV by RT-qPCR

		JEV RT-qPCR Results, Cq Value													
		140 µL							5 mL, Microcentrifuge						
		Extract 1		Extract 2		Extract 3			Extract 1		Extract 2		Extract 3		
		1	2	1	2	1	2	Mean	1	2	1	2	1	2	Mean
Baseline, t0															
Neat	High	27.49	ND	27.56	ND	27.29	ND	27.45							
	Medium	31.10	ND	31.47	ND	31.22	ND	31.27							
	Low	35.51	ND	35.27	ND	33.85	ND	34.87							
Stored for 6 h at 4°C															
Neat	High	30.25	30.7	31.02	30.67	30.29	33.14	31.01	30.64	30.01	29.98	30.11	30.26	30.1	30.18
	Medium	34	35.6	35.92	34.95	35.3	34.05	34.97	32.64	34.68	35.09	33.52	33.85	34.83	34.1
	Low	Neg	Neg	Neg	Neg	Neg	Neg	Neg	Neg	Neg	Neg	Neg	Neg	Neg	Neg
Stored for 4 d at –80°C															
Neat	High	27.95	28.03	29.57	28.98	29.08	27.68	28.55	31.73	30.7	31.61	30.31	ND	ND	31.08
	Medium	32.62	31.85	33.38	32.47	32.1	30.25	32.11	31.71	31.68	33.71	32.18	ND	ND	32.32
	Low	Neg	37.78	36.07	33.75	38.6	39.32	Neg^a^	Neg	Neg	Neg	Neg	ND	ND	Neg
With buffer AVL	High	27.17	27.46	26.79	26.69	26.36	26.84	26.88	25.95	25.92	26.06	25.75	ND	ND	25.92
	Medium	30.83	30.6	31.02	30.19	30.32	30.04	30.50	28.86	28.44	28.99	29	ND	ND	28.83
	Low	36.24	34.11	35.35	36.4	34.18	35.23	35.25	33.33	33.42	33.36	33.78	ND	ND	33.47
Stored for 14 d at –80°C															
Neat	High	32.03	31.78	28.41	29.07	28.46	28.59	29.72							
	Medium	32.78	32.67	34.63	35.75	33.25	34.11	33.87							
	Low	Neg	Neg	Neg	38.22	Neg	Neg	Neg^a^							
With buffer AVL	High	26.91	26.62	26.56	26.74	26.88	26.86	26.76							
	Medium	30.93	31.1	30.03	29.74	29.87	29.9	30.26							
	Low	35.1	34.62	35.58	34.4	33.79	33.9	34.56							
Stored for 30 d at –80°C															
Neat	High	32.13	ND	32.76	ND	ND	ND	32.44							
	Medium	37.16	ND	36.03	ND	35.13	ND	36.10							
	Low	Neg	ND	Neg	ND	38.70	ND	Neg^a^							
With buffer AVL	High	30.76	ND	30.38	ND	30.85	ND	30.66							
	Medium	30.08	ND	34.25	ND	33.65	ND	32.66							
	Low	39.13	ND	37.66	ND	37.54	ND	38.11							

“T0” indicates extracted and tested immediately after virus spiking and preparation of dilutions. Three extraction replicates were performed, on the same run, and 2 RT-qPCR replicates for each extraction. “Controls” indicates PCR-positive controls (x2 JEV G3) and negative controls (no template) in duplicate positive and negative, respectively, MS2 extraction positive control positive for each sample. “ND” indicates not done. This was in an effort to conserve reagents and microcentrifuge kits. Cq >40 were reported as negative and ≤40 as positive.

Abbreviations: JEV, Japanese encephalitis virus; RT-qPCR, reverse transcription real-time polymerase chain reaction.

^a^Mean reported as negative if 1 or more extracts were negative.

### Patient Samples

#### Retrospective Study

Twenty-four patients with suspected encephalitis, recruited between 2014 and 2017, were tested. Five (21%) were anti-JEV MAC-ELISA positive in CSF and serum (Table 3). JEV RNA was not detected by RT-qPCR in any (0/24) of the urine samples of the patients, or in the corresponding throat, CSF, or serum samples. MS2 phage as an internal control was detected by RT-qPCR in all samples with the expected Cq value.

**Table 3.** JEV MAC-ELISA and JEV RT-qPCR Results for Patient Samples

			JEV MAC-ELISA Result (ISR)^a^			JEV RT-qPCR Result (Cq Value)							
				ConvaleScent		Throat Swab		Urine		CSF		Serum	
Patients	Age, y	Sex	Acute Serum	Serum	CSF	NS2A	NS3	NS2A	NS3	NS2A	NS3	NS2A	NS3
A, Retrospective study													
1	42	M	Neg (4.9^b^)	-	Neg (5.2^b^)	-	-	Neg	Neg	Neg	-	Neg	-
2	0.5	M	Neg (1.7)	-	Neg (1.4)	Neg	Neg	Neg	Neg	Neg	-	-	-
3	0.7	F	Neg (1.3)	-	Neg (1.2)	Neg	Neg	Neg	Neg	-	-	-	-
4	20	F	Neg (1.3)	-	Neg (1.3)	-	-	Neg	Neg	Neg	-	Neg	-
5	40	F	Neg (1.4)	-	Neg (1.3)	-	-	Neg	Neg	Neg	-	Neg	-
6	20	M	Neg (1.1)	Neg (1.3)	Neg (1.6)	-	-	Neg	Neg	Neg	-	Neg	-
7	22	F	Neg (1.4)	-	Neg (0.5)	-	-	Neg	Neg	Neg	-	Neg	-
8	17	F	Neg (5.7)	Pos (13.3)	Pos (39.8)	-	-	Neg	Neg	Neg	Neg	-	Neg
9	2	F	Neg (2.2)	-	Neg (2.4)	-	-	Neg	Neg	-	-	-	-
10	32	M	Neg (1.6)	Neg (1.6)	Neg (2.7)	-	-	Neg	Neg	Neg	-	Neg	-
11	19	M	Pos (18.4)	Pos (45.1)	Pos (29.8)	Neg	Neg	Neg	Neg	Neg	Neg	Neg	Neg
12	50	M	Pos (26.0)	Pos (40.8)	Pos (34.2)	Neg	Neg	Neg	Neg	Neg	Neg	Neg	Neg
13	0.3	F	Neg (1.6)	Neg (2.4)	Neg (1.7)	Neg	Neg	Neg	Neg	Neg	Neg	-	-
14	13	M	Neg (1.4)	Neg (1.8)	Neg (2.2)	Neg	Neg	Neg	Neg	-	-	-	-
15	3	F	Neg (1.4)	Neg (1.1)	Neg (1.9)	Neg	Neg	Neg	Neg	-	-	-	-
16	23	M	Pos (6.3)	Neg (4.8)	Pos (6.4)	Neg	Neg	Neg	Neg	-	-	-	-
17	0.6	F	Neg (1.1)	Neg (1.1)	Neg (1.3)	Neg	Neg	Neg	Neg	-	-	-	-
18	4	F	Neg (1.1)	-	Neg (0.7)	Neg	Neg	Neg	Neg	-	-	-	-
19	1.5	M	Neg (1.2)	Neg (1.2)	Neg (1.3)	Neg	Neg	Neg	Neg	-	-	-	-
20	38	M	Neg (1.1)	Neg (1.3)	Neg (1.2)	Neg	Neg	Neg	Neg	Neg	Neg	-	-
21	13	M	Pos (11.3)	Pos (27.9)	Pos (60.3)	Neg	Neg	Neg	Neg	Neg	Neg	-	-
22	14	M	Pos (21.7)	-	Pos (38.2)	Neg	Neg	Neg	Neg	Neg	Neg	Neg	Neg
23	0.2	F	Neg (1.3)	Neg (1.3)	Neg (1.3)	Neg	-	Neg	Neg	-	-	-	-
24	0.1	M	Neg (1.4)	-	Neg (1.3)	-	Neg	Neg	Neg	-	-	-	-
B, Prospective study													
1	13	M	Pos (22.3)	-	Pos (42.8)	Pos (36)	-	Neg	-	Neg	-	Neg	-
2	34	M	Neg (1.8)	-	Neg (2.8)	Neg	-	Neg	-	Neg	-	Neg	-
3	6	F	Pos (6.4)	-	Pos (40.3)	Neg	-	Neg	-	Neg	-	-	-
4	3.8	M	Pos (32.6)	-	Pos (48.5)	-	-	Neg	-	Neg	-	Neg	-
5	24	M	Pos (18)	-	Pos (41.8)	Neg	-	Neg	-	Neg	-	Neg	-
6	18	M	Neg (1.2)	-	Neg (1.3)	Neg	-	Neg	-	Neg	-	Neg	-
7	35	M	Pos (17.5)	-	Pos (46.8)	Neg	-	Neg	-	Neg	-	Neg	-
8	3	M	Pos (22.4)	-	Pos (49.9)	Neg	-	Neg	-	Neg	-	Neg	-
9	27	M	Neg (1.3)	-	Neg (1.3)	Neg	-	Neg	-	Neg	-	Neg	-
10	45	M	Neg (1.3)	-	Neg (1.3)	Neg	-	Neg	-	Neg	-	Neg	-
11	18	M	Neg (1.5)	-	Neg (3.1)	Pos (32)	-	Neg	-	Neg	-	Neg	-
12	25	F	Neg (1.5)	-	Neg (1.4)	Neg	-	Neg	-	Neg	-	Neg	-
13	41	F	Neg (1.1)	Neg (1.4)	Neg (1.4)	Neg	-	Neg	-	Neg	-	Neg	-
14	16	F	Neg (1.1)	-	Neg (1.4)	Neg	-	Neg	-	-	-	Neg	-
15	25	M	Neg (0.9)	-	Neg (0.6)	Neg	-	Neg	-	-	-	-	-
16	55	F	Neg (5.3)	-	Neg (1.3)	Neg	-	Neg	-	Neg	-	Neg	-
17	26	F	Neg (1.6)	-	Neg (1.4)	Neg	-	Neg	-	Neg	-	Neg	-

Results from testing throat samples, CSF, and serum, but not urine, have previously been published [9]. “Pos” indicates anti-JEV IgM positive. “Neg” indicates anti-JEV IgM negative or equivocal, with ISR cutoffs calculated according to the manufacturer’s instructions. RT-qPCR “Neg” indicates no amplification curve or curve with a Cq >40. RT-qPCR “Pos” indicates amplification curve with Cq ≤40. Hyphen indicates that no sample was available for testing. NS2A and NS3 are the 2 RT-qPCR assays used for testing, targeting the respective segments of the genome [25]. Positive RT-qPCR results were confirmed by sequencing.

Abbreviations: CSF, cerebrospinal fluid; ISR, immune status ratio; JEV, Japanese encephalitis virus; MAC-ELISA, anti-JEV IgM capture enzyme-linked immunosorbent assay; RT-qPCR, reverse transcription real-time polymerase chain reaction.

^a^Anti-JEV IgM detection using the JE Detect IgM Antibody Capture ELISA Kit (InBios).

^b^Indicates that a different kit was used for MAC-ELISA testing, as per the laboratory protocol at that time (Cat. No. E-JED01C; Inverness Medical Innovations [formerly Panbio Ltd.], Brisbane, Australia).

#### Prospective Study

Seventeen patients with suspected encephalitis, recruited between June and December 2017, were tested. Six (35%) were found anti-JEV MAC-ELISA positive in CSF and serum (Table 3). Results from the evaluation (see “Evaluation of Urine Preparation and Storage”) showed that addition of lysis buffer to fresh urine before freezing improved the preservation of JEV for subsequent detection by RT-qPCR. Therefore, for these patients, 100 µL of buffer ATL carrier RNA was added to 300 µL of urine before storage at –80°C until testing (3–7 days later). The EZ1 extraction kit protocol involves adding 1:4 buffer ATL, so the samples were frozen with this volume of ATL carrier RNA.

JEV RNA was not detected by RT-qPCR in any (0/17) of the urine samples of the patients, or in the available corresponding CSF or serum samples. MS2 phage as an internal control was detected by RT-qPCR with an expected Cq value in all samples. JEV RNA was detected by RT-qPCR from throat swab in 2 patients, as recently reported [9].

## DISCUSSION

A key finding of this study was the degradation of JEV RNA in urine even for short storage periods at 4°C or –80°C. Therefore, to improve the detection of JEV in urine, we investigated the effects of, on the 1 hand, virus concentration using a Microsep device, and, on the other hand, the addition of lysis buffer (AVL or ATL) in fresh urine before storage to play the role of a nucleic acid stabilizer.

Our findings suggest that concentration using a Microsep Advance centrifugal device did not improve detection of JEV RNA in urine without AVL. However, there was possibly a slight synergistic effect with AVL, generating marginally lower Cq values at all dilutions. Microsep devices have been shown to concentrate nonenveloped RNA viruses [25]; however, the lack of improvement at the limit of detection of a flavivirus, dengue, in patients’ urine samples has been reported [26]. This may be due to loss of virus by adsorption onto the membrane or other part of the device. Notably, a recent study highlighted the presence of small viral RNA fragments in urine and the successful application of ultrafiltration devices in conjunction with PCR assays especially designed to detect small amplicons [27].

In contrast to our findings using an ultrafiltration device, we showed that the addition of buffer AVL carrier RNA to fresh urine permitted stabilization of viral RNA for subsequent detection by RT-qPCR even after a freezing step. Optimal detection of low viral titer requires immediate processing of fresh samples, which is often difficult to implement. We showed that this constraint can be easily overcome with a simple addition of buffer AVL carrier RNA to fresh samples, permitting the deferment of molecular assays without losing analytical sensitivity during storage. Similar findings of the improved detection of Zika virus RNA in urine by storage with a nucleic acid stabilizer have been recently demonstrated [28]. Moreover, the addition of lysis buffer would potentially lead to the release of the cellular JEV RNA present in infected cells, and the subsequent preservation of the JEV RNA at –80°C. It is acknowledged that only AVL and ATL buffers were evaluated in the experimentation, and other stabilizers such as Urine Conditioning Buffer (Zymo Research Corp.) may prove superior to AVL/ATL, as recently reported [29]. However, we used lysis buffer from Qiagen extraction kits, which are widely distributed and utilized worldwide. For this reason, the method has the advantage that it does not require the use of additional reagents.

However, JEV was not detected in the urine of the 41 AES patients that we tested in our pilot study. Those patients were recruited as part of the South-East Asia Encephalitis study and fulfilled clinical criteria for encephalitis. They had onset of symptoms within 7 days and received comprehensive laboratory investigations. Eleven (27%) were positive by the WHO reference standard test for JEV (JEV MAC-ELISA), 5/24 (21%) in the retrospective study and 6/17 (35%) in the prospective study. Two (5%) were positive by JEV RT-qPCR testing, both from throat swab only, as previously reported [9].

There is a paucity of existing literature on this topic; nevertheless, the finding of no JEV RNA detected in urine is consistent with a previous study of 52 patients in China [17]. However, 2 recent individual case reports have described detection of JEV RNA in the urine of 2 patients with anti-JEV IgM detected by MAC-ELISA in CSF and serum [18, 19]. The first report, by Mai et al., demonstrated JEV RNA detection in urine on day 3 of admission (approximately the fourth day from onset of symptoms), and not in another urine sample from day 15 of admission. In comparison, the second report, by Huang et al., detected JEV RNA in urine first on day 9 of admission (approximately the 14th day from onset of symptoms), and on repeat testing up to day 20 of admission. All patients in the present study were sampled within 7 days of onset of symptoms. Additionally, the 2 case reports were performed on fresh urine specimens, whereas the 24 retrospective samples in this study were stored at –80°C for up to 3 years before our investigation. Only 17 patients from the prospective study were stored in buffer ATL carrier RNA, permitting similar sensitivity of detection by RT-qPCR to that observed using fresh urine. RNA in urine may be particularly sensitive to degradation, as urine contains RNases with activity as much as 100-fold higher than in serum [28].

Limitations of the study include the limited number of replicates and small number of samples stored in optimal conditions. It is notable that, although 11 patients were positive by JEV MAC-ELISA, only 2 patients were confirmed as having JEV infection, and for this reason, the inference that urine is a poor sample for the detection of JEV infection may be unreliable, as it is based on only a very small sample of patients [8]. The optimization experiments were performed with cell culture supernatant to provide a model of JEV in an infected patient’s urine sample; however, it is recognized that the abundance of RNA as compared with intact virus may not be the same as that in clinical infection. It would have been useful to have tested additional time points between 0 and 6 hours to determine the rapidity of RNA degradation in urine more precisely. In addition, patients’ urine samples were only tested at 1 time point, and this was not the same for all patients studied.

The fact that JEV was not detected in urine for the 11 JEV MAC-ELISA–positive patients of this study, in the context of the 3 other publications [17–19], suggests that excretion of JEV RNA may be brief and intermittent, and possibly that human kidney cells are not a site of high levels of replication. Ricklin et al. demonstrated that pigs, the amplifying host for JEV, do not demonstrate viruria, but instead that the tonsils are a site of high replication [30]. An in vitro study of replication of JEV in various cell types could be used to explore the findings further.

JEV diagnostics, as with many flaviviruses associated with neurological syndromes, are based on serological tests that are notoriously difficult to interpret. Although molecular diagnosis is invaluable, JEV RNA is only rarely detected in serum or CSF. Urine is relatively easy to obtain and plentiful. There has been minimal investigation into the role of testing urine for JEV RNA to date. Our study suggests that JEV RNA is not easily detected in urine; however, it does demonstrate the value in storage of urine with a nucleic acid stabilizer at the point of bedside collection. This suggests a role for larger rigorous studies testing urine for JEV RNA, with urine collected at different times from symptom onset, and using buffer AVL carrier RNA or a similar nucleic acid stabilizer [29] for immediate storage of fresh urine.

## Supplementary Data

Supplementary materials are available at *Open Forum Infectious Diseases* online. Consisting of data provided by the authors to benefit the reader, the posted materials are not copyedited and are the sole responsibility of the authors, so questions or comments should be addressed to the corresponding author.
